# Repeated Copulation and Guarding, and Their Relationship With Male and Female Morphological Traits in the Water Scorpion *Nepa hoffmanni*

**DOI:** 10.1002/ece3.70725

**Published:** 2024-12-23

**Authors:** Hyoseul Hyun, Byeongho Lee, Chang S. Han

**Affiliations:** ^1^ Department of Biology Kyung Hee University Seoul Korea; ^2^ School of Biological Sciences Monash University Melbourne Victoria Australia; ^3^ Korea Institute of Ornithology Kyung Hee University Seoul Korea

**Keywords:** mate‐guarding, mating behaviour, *Nepa hoffmanni*, Nepomorpha, repeated copulation, water scorpion

## Abstract

Insects copulate multiple times not only with different mates but also with the same mate, which is called repeated copulation. It occurs as a repeated alternation between copulation and mate‐guarding, leading to the prolonged physical attachment between males and females. Particularly, in species where males forcefully grasp females, attempt to mate without courtship and exhibit repeated copulations, male and female morphological traits are expected to be associated with mating characteristics. In this study, we describe for the first time the detailed mating behaviour and patterns of repeated copulations in the water scorpion *Nepa hoffmanni* (Nepidae, Hemiptera). *Nepa hoffmanni* repeated copulation and guarding approximately 10 times on average. Over repeated copulations, copulation duration decreased while guarding duration increased, potentially due to decreased male sperm reserves and increased female mating reluctance. Additionally, we found that average guarding duration was positively associated with male leg length and negatively associated with female leg length. This suggests that shorter male legs may enhance courtship efficiency or intensity, while shorter female legs may be less effective at resisting male mating attempts, leading to a quicker initiation of subsequent copulations with the same partner. This indicates that the evolution of repeated copulations may be linked to the evolution of morphological characteristics. Therefore, our study provides novel insights into the evolution of the mating behaviour of water scorpions.

## Graphical abstract

We describe for the first time the detailed mating behaviour and patterns of repeated copulations in the water scorpion *Nepa hoffmanni* (Nepidae, Hemiptera). Over repeated copulations, copulation duration decreased while guarding duration increased. Additionally, we found that average guarding duration was positively associated with male leg length and negatively associated with female leg length.

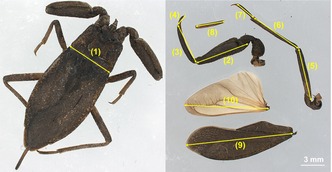

## 1 Introduction

In many insects, multiple copulations with different opposite‐sex partners are common (Arnqvist and Nilsson 2000; Ridley 1988). However, some insects stay with their opposite‐sex mating partners after copulation (i.e., post‐copulation mate‐guarding) and repeatedly copulate with the same partner over an extended period. This mating behaviour is referred to as ‘repeated mating’ or ‘repeated copulation’ (Hunter et al. 1993). Despite the apparent advantages associated with multiple copulations with different partners (Arnqvist and Nilsson 2000), several hypotheses have been proposed to explain the adaptive benefits from repeated copulation (Choe 1997; Hunter et al. 1993). For instance, in the cricket *Ornebius aperta* where females rapidly consume spermatophores, males increase total sperm transfer through repeated copulation while decreasing the number of sperm in a single spermatophore (Laird, Gwynne, and Andrade 2004). In addition, in species exhibiting strong last male sperm precedence, males can achieve a high level of paternity assurance through repeated copulations (e.g., giant water bugs *Abedus herberti* (Smith 1979); fly *Dryomyza anilis* (Otronen 1994)). Repeated copulation may also be favoured in situations where the likelihood of encountering multiple mates throughout a lifetime is low (Andrade and Banta 2002).

Repeated copulation is likely to be associated with prolonged mate‐guarding. In species exhibiting mate‐guarding, males physically attach to females after copulation, either through direct contact (contact mate‐guarding) or by maintaining close proximity to females (non‐contact mate‐guarding) (Alcock 1994; Simmons and Siva‐Jothy 1998). As repeated copulation involves an alternation between copulation and mate‐guarding, an increased frequency of copulations with the same female results in prolonged contact or non‐contact mate‐guarding. Post‐copulation mate‐guarding serves multiple functions, providing the male not only with opportunities for repeated insemination but also influencing the control of the female to use his sperm for fertilisation (Simmons 2002). In particular, in insect species exhibiting repeated copulations, mate‐guarding can be regarded not only as a post‐copulation strategy to win at sperm competition but also as a pre‐copulation strategy to secure access to mates. Therefore, in those insects, reproductively successful males are likely to be those who engage in numerous copulations with the same female and guard her for an extended duration.

Furthermore, in species engaging in sexual coercion and repeated copulation with contact mate‐guarding, it is expected that the morphological traits of both sexes are linked to mating characteristics, such as copulation frequency, copulation duration, and guarding duration. In species exhibiting coercive mating, male morphological traits related to overcoming female resistance allow males to sustain prolonged mate‐guarding and engage in more frequent copulation with the same female. Females with morphological traits that are advantageous for resisting excessive mating with the same male may exhibit shorter copulation and guarding durations, resulting in a lower frequency of copulation. For example, the mating behaviour of the water strider species *Gerris gracilicornis* features repeated copulation, prolonged mate‐guarding, and forceful male mating attempts (Han et al. 2010; Han and Jablonski 2009). When a male mounts a female for mating, he tightly grasps the female thorax with his forelegs while simultaneously attaching his genitalia to the female genital segment (Han et al. 2010). Notably, *G. gracilicornis* males with a body length 86% shorter than that of females were theoretically found to produce the greatest grasping force for overcoming female resistance (Han et al. 2010), and empirical studies confirmed their greater mating success (Han and Jablonski 2018; Han et al. 2010). This finding suggested that morphological features play a crucial role in determining the mating characteristics associated with repeated copulation. Consequently, in species engaging in sexual coercion and repeated copulation with contact mate‐guarding, mating characteristics are expected to be influenced by morphological traits in both sexes.

Our study presents the first detailed description of mating behaviours, repeated copulations and mate‐guarding in the water scorpion species *Nepa hoffmanni* (Nepidae, Hemiptera). We also assessed the sex‐specific relationships between their mating behaviours and morphological traits. Like the other Nepidae species, *N. hoffmanni* spends most of its adult life submerged, even during mating (Ban, Shibata, and Ishikawa 1988). Previous studies on Nepidae mating have mainly concentrated on the early phase, starting from male mounting of the female to the intromission of genitalia (Hamilton 1931; Keffer and McPherson 1993; Packauskas and McPherson 1986; Tawfik and Awadallah 1975), and descriptions of Nepidae post‐copulatory mating behaviour are scarce (Bailey 1984; Jayakumar and Mathavan 1985; Venkatesan and Ravisankar 1993). For example, *Laccotrephes griseus* males are known to exhibit repeated copulation and prolonged mate‐guarding (even up to 2 days) (Jayakumar and Mathavan 1985; Venkatesan and Ravisankar 1993). Similarly, in *Ranatra dispar* , up to 3 separate copulations were observed within an hour (Bailey 1984).

**FIGURE 1:**
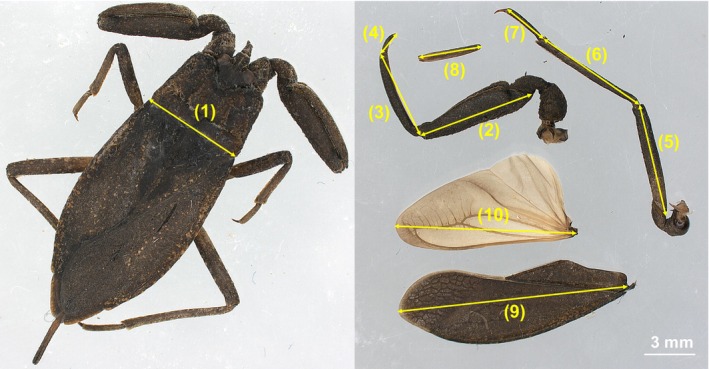
Morphological measurements of *Nepa hoffmanni* . (1) Pronotum width, (2) foreleg femur length, (3) foreleg tibia length, (4) foreleg tarsus length, (5) hindleg femur length, (6) hindleg tibia length, (7) hindleg tarsus length, (8) siphon length, (9) forewing length, and (10) hindwing length.

We documented the mating behaviour of 14 pairs of *N. hoffmanni* from the initial recognition between a male and a female to the moment when the male dismounted after an alternation of copulation and contact mate‐guarding. We measured the copulation frequency and duration of each copulation and guarding, and calculated the total duration of copulations and guarding. We also broke the mating of *N. hoffmanni* down into several distinct stages (e.g., mounting and aligning, copulation, male genital withdrawal, guarding) with detailed descriptions of the behaviour exhibited by both sexes in each stage. Moreover, we explored the relationships between mating characteristics (copulation frequency, duration of each copulation and guarding, and total duration of copulations and guarding) and morphological traits (refer to Figure 1) in both males and females.

Given our findings that *N. hoffmanni* males grasp females using their forelegs and stimulate females using both forelegs and hindlegs (see Results), we expected a positive association between the lengths of male forelegs or hindlegs and mating characteristics. Additionally, as *N. hoffmanni* females lifted their bodies and moved around during the guarding period, potentially disturbing the males' stable grasp (see Results), we also predicted that female leg components would be associated with mating characteristics.

## 2 Materials and Methods

### 2.1 Study Species and Rearing Conditions

*Nepa hoffmanni* is an aquatic insect species widely distributed throughout East Asia (Korea, Japan, China and Russia) (Ban, Shibata, and Ishikawa 1988). *Nepa hoffmanni* follows a univoltine life cycle: adults overwinter from December to March (reproductive diapause) and become reproductively active from April to May (Ban, Shibata, and Ishikawa 1988). The summer generation that emerges from the overwintered generation does not reproduce in the same year but engages in reproduction the following year after the winter diapause (Ban, Shibata, and Ishikawa 1988). Notably, some individuals are known to live for up to 3 years, reproducing annually after each diapause (Ban, Shibata, and Ishikawa 1988).

We collected 14 adult males and 14 adult females from springs and streams in Wanju‐gun, Jeollabuk‐do, Korea in May 2021. We transported them to a climate room at Kyung Hee University, Korea, and maintained them at 25°C ± 2°C under a 17L:7D photoperiod. Each individual was housed individually in rearing dishes (diameter × height:10 × 4 cm, water depth: 1 cm) and provided with two pieces of styrofoam (3 × 3 × 0.3 cm) for refuge. Regular maintenance included cleaning the rearing dishes, refreshing water, and providing food (cricket *Gryllus bimaculatus*) three times per week. To facilitate reproductive activity and an eagerness to mate, individuals underwent a 1‐month period of individual rearing before being subjected to mating assays.

### 2.2 Mating Assays

We placed one male and one female in a dish (diameter × height:10 × 4 cm, water depth: 1 cm) and recorded their mating behaviour. To identify male genitalia intromission, recordings were focused on the ventral side of pairs. Given the lack of prior descriptions of *N. hoffmanni* mating behaviour, we comprehensively documented and described all male and female behaviours throughout mating. The occurrence of behaviours at each mating stage (e.g., mounting and aligning, copulation, male genital withdrawal, guarding) was systematically measured.

### 2.3 Morphological Measurements

We estimated the length of multiple morphological traits for each individual at the 1 μm scale using a light microscope after death (Leica S9D). The measured traits included: (1) Pronotum width, (2) Foreleg femur length, (3) Foreleg tibia length, (4) Foreleg tarsus length, (5) Hindleg femur length, (6) Hindleg tibia length, (7) Hindleg tarsus length, (8) Siphon length, (9) Forewing length, and (10) Hindwing length (Figure 1). The total foreleg and hindleg lengths were calculated by summing their respective components (Figure 1).

We measured these traits because we predicted that they affect reproductive characteristics such as copulation or guarding duration or frequency. Pronotum width was used as an indicator of body size, whereas leg components were measured because male forelegs and hindlegs play a role in courtship, and female hindlegs are involved in physical resistance. Siphon and wing lengths were included due to their importance in underwater respiration of Nepidae (Hamilton 1931; Parsons 1972).

### 2.4 Statistical Analyses

#### 2.4.1 Repeated Copulation and Guarding

We constructed a univariate linear mixed‐effect model to test how copulation or guarding duration changed with repeated copulations or guardings. In this model, we fitted copulation duration or guarding duration as a response variable, the number of repetitions as a covariate, and the pair ID as a random effect. In addition, to test whether the final guarding duration differed from the duration of preceding guardings, we built another univariate linear mixed‐effect model. In this model, guarding duration was the response variable and sequence (a two‐level factor: final guarding or not) was included as a fixed effect. The pair ID was fitted as a random effect in this model.

The significance of random effects was evaluated through a likelihood ratio test (LRT). We calculated the likelihood ratio as twice the difference in log likelihood between models with or without the focal random effect. We obtained a *p* value from the LRT, assuming 1degree of freedom. All models were tested using ASreml 4.2 software (VSN Interaction, Hemel Hempstead, UK).

### 2.5 Correlations Between Copulation Duration and Guarding Duration and Morphological Traits

Since mating behaviours were repeatedly measured at the individual level, we could calculate among‐individual correlations between mating behaviours and morphological traits using bivariate mixed‐effects models. However, due to the small sample size (14 males and 14 females), we instead used Spearman's rank correlation coefficient to explore associations between log‐transformed relative morphological traits and individual‐specific average mating behaviours. The relative lengths of morphological traits (e.g., total foreleg length, foreleg femur length, foreleg tibia length, foreleg tarsus length, total hindleg length, hindleg femur length, hindleg tibia length, hindleg tarsus length, siphon length, forewing length, and hindwing length) were calculated by dividing these lengths by the pronotum width, a recognised body size indicator in previous Hemiptera studies (Joseph et al. 2016; Miller and Emlen 2010; Eberhard 1998; McLain, Lanier, and Marsh 1990; Larsson 1989). Subsequently, the relative morphology lengths underwent a log transformation.

For each mating pair, we calculated five mating behaviours: (1) total copulation duration (sum of copulation duration over repetitions), (2) total guarding duration (sum of guarding duration over repetitions), (3) copulation frequency, (4) average copulation duration (total copulation duration divided by copulation frequency), and (5) average guarding duration (total guarding duration divided by copulation frequency). Spearman's rank correlation coefficients were calculated using STATISTICA software (version 14).

## 3 Results

### 3.1 Mating Sequence

#### 3.1.1 Mounting and Aligning (Video S1)

When a male *N. hoffmanni* attempts to copulate, he clasps the body or legs of a female with his foreleg and mounts her without apparent courtship behaviours. Subsequently, the male turns around on the female's back to align the genitalia in the same direction as the female's. When the mounting is successful, he then grasps the female's thorax or the side of her abdomen and twists his abdomen downwards, positioning his genitalia underneath the female's hindmost abdominal segment. Among successful copulation attempts, 79% (11 of 14) of males twisted their abdomen in only one direction (left or right side from the ventral view) during repeated copulations; specifically, 9 males twisted only the left side, and 2 males twisted only the right side. The other 3 males did not prefer a particular direction when they twisted their abdomens.

#### 3.1.2 Male Genitalia Extension (Video S2)

**FIGURE 2:**
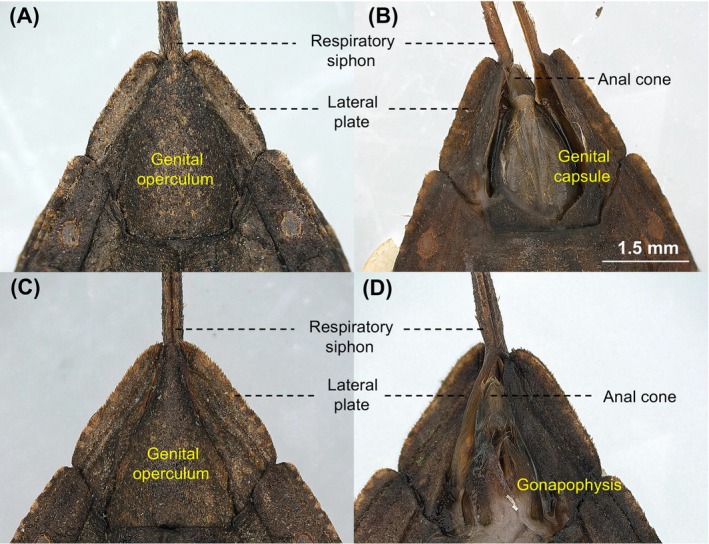
Light microscope image of the final abdominal segment of male (A, B) and female (C, D) *Nepa hoffmanni* . The genital operculum was dissected in (B) and (D).

While twisting the abdomen, the male opens the genital operculum, separating two lateral plates connected to each strap of the respiratory siphon (Figure 2). The genital capsule, representing the male internal genitalia, is then extended from the opening of the abdominal segment. The male inserts the genital capsule into the opening of the female genitalia operculum. If the female does not open her operculum, the male cannot insert his genital capsule and retracts his genitalia, remaining in the pre‐copulatory guarding state. Notably, during this phase, no apparent resistant behaviours of females toward males were observed. Approximately 43% of the males (6 of 14) failed the first copulation attempt, entering the pre‐copulatory guarding phase.

#### 3.1.3 Copulation

Copulation duration, from successful intromission to male genitalia withdrawal, encompasses sperm transfer through the insertion of the genital capsule into the gonapophysis. The average duration of the initial successful copulation was 12.28 min (SE = 10.77, min = 1.03 min, max = 47.07 min, *N* = 14).

#### 3.1.4 Male Genitalia Withdrawal

After copulation, the male unfolds the twisted abdomen, detaches the genital capsule from the female genitalia, and withdraws it into his genital operculum.

#### 3.1.5 Guarding

Following the first copulation, the male engages in post‐copulatory contact mate‐guarding, remaining on the female's back without genital attachment. Guarding duration is defined as the time from genital capsule detachment to either another intromission (Video S3) or the male's dismount. After the first copulation, guarding persisted for an average of 13.21 min (SE = 30.01, min = 0.55 min, max = 110.9 min, *N* = 14). Additionally, in our observation, females were capable of expelling sperm from their reproductive tract into the water immediately after copulation (Video S3).

### 3.2 Male and Female Mating Behaviours Occurred in Each Mating Sequence

We categorised one female mating behaviour and two male mating behaviours observed during the pre‐copulation, copulation, and post‐copulation phases (Table 1). Each behaviour is defined in Table 1. We also calculated the proportion of individuals exhibiting specific mating behaviours at each sequence (Table 2). Below, we detail the expression of each mating behaviour across these sequences. All the male and female behaviours described below occurred irregularly during mating.

**TABLE 1:** The mating stage and mating behaviour of male and female *Nepa hoffmanni*.

	Definition
Stage	
Mounting and aligning	The period from when a male mounts until his genitalia is extended. During this phase, the male aligns his body with the female
Male genitalia extension	Behaviour where the male extends his genitalia and inserts it into the female's genital operculum
Copulation	The phase from male genital intromission until the male genital capsule detaches from the female genital operculum
Male genitalia withdrawal	Behaviour where the male withdraws the genital capsule into his genital operculum after detaching from the female genitalia
Guarding	The phase from the detachment of male and female genitalia to the subsequent male genitalia extension. During this phase, the male remains on the female's back without genital intromission
Behaviour	
Female	
Uplift	Behaviour where a female raises her abdomen using hindlegs, exposing the male to the water surface
Male	
Tapping female thorax/abdomen	Behaviour where a male taps the thorax or abdomen of a female with forelegs or midlegs
Male	
Hindleg vibration	Behaviour involving the vibration of at least one hindleg

**TABLE 2:** The proportion of individuals who exhibited a certain mating behaviour in each mating sequence.

	Mounting and aligning	Male genitalia extension	Copulation	Male genitalia withdrawal	Guarding
Female (*n* = 14)					
Uplift			14% (*n* = 2)		21% (*n* = 3)
Male (*n* = 14)					
Tapping female thorax/abdomen	7% (*n* = 1)	71% (*n* = 10)	79% (*n* = 11)		71% (*n* = 10)
Hindleg vibration	21% (*n* = 3)	57% (*n* = 8)	50% (*n* = 7)	43% (*n* = 6)	57% (*n* = 8)

#### 3.2.1 Mounting and Aligning

Males exhibited no typical behaviours during this phase. However, among 14 males, 4 males expressed behaviours such as hindleg vibration (*N* = 3) and tapping the female's thorax/abdomen with forelegs (*N* = 1) (Table 2). In females, no resistance behaviours were observed during the mounting and aligning phase.

#### 3.2.2 Male Genitalia Extension

In males, 71% (10 of 14) tapped the female's thorax/abdomen with forelegs when they extended their genitalia for copulation (Table 2). During this phase, 57% (8 of 14) exhibited hindleg vibration (Table 2). Females did not exhibit any apparent behaviours during this phase.

#### 3.2.3 Copulation

Tapping the female's thorax/abdomen with the forelegs was exhibited by 79% of the males (11 of 14) within a minute after intromission (Table 2). Hindleg vibration was observed in 50% of the males (7 of 14) during copulation (Table 2). Females did not exhibit any apparent behaviours during the early period of copulation (within a minute after intromission) (Table 2). However, during copulation, two females lifted their abdomens, exposing the mounted males above the water surface, which lasted between 9 s and 8.3 min.

#### 3.2.4 Male Genitalia Withdrawal

Within the minute before genitalia withdrawal, 43% of the males (6 of 14) vibrated their hindlegs. No noteworthy behaviours were noted in females during this phase.

#### 3.2.5 Guarding

During the guarding periods, 71% of the males (10 of 14) tapped females with their forelegs, and 57% (8 of 14) vibrated their hindlegs. In contrast, 21% of the females (3 of 14) were observed lifting a male up for durations of 5.71 min, 6 s, and 1 s, respectively.

### 3.3 Repeated Copulation and Guarding

We observed that *N. hoffmanni* males engage in multiple copulations and contact mate‐guarding while holding females. After the first copulation, males remain on the female's back (i.e., post‐copulation contact mate‐guarding). After a few minutes of guarding, males twist their abdomen, expand the genital capsule, and attempt another intromission. All pairs, except one, engaged in a second copulation after the first guarding period. In the one pair where repeated copulation did not occur, the male attempted to solicit copulation through tapping and vibrations, but the female did not open her genital operculum, leading the male to voluntarily dismount. This suggests that all *N. hoffmanni* males attempt repeated copulations and achieve copulation if the female consents.

**FIGURE 3:**
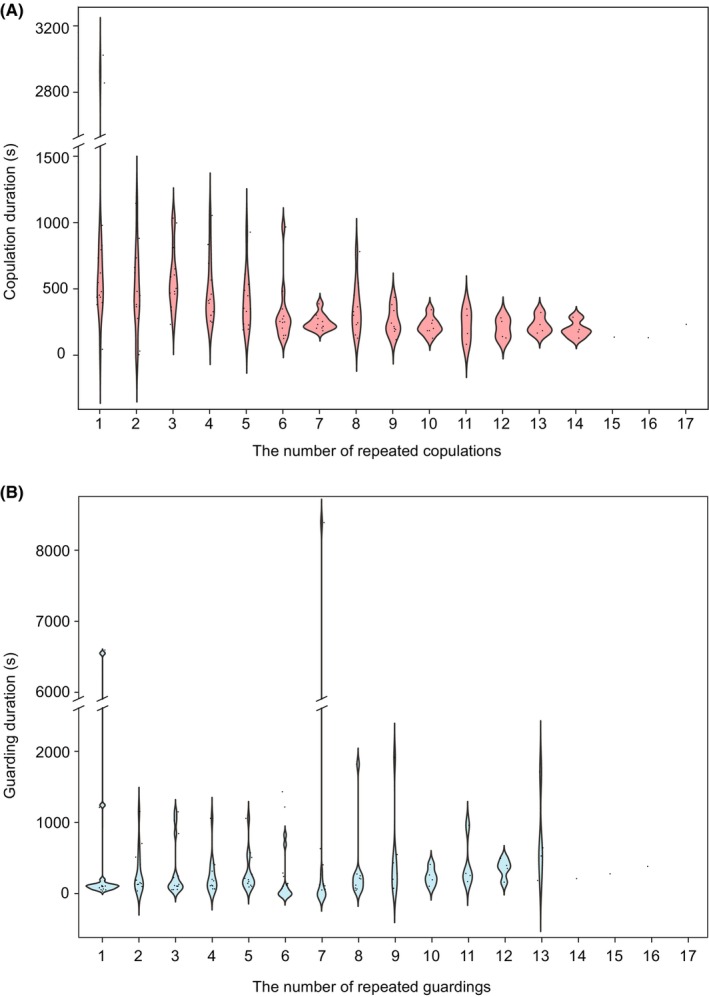
Changes in (A) copulation and (B) guarding durations during repeated copulations characterised by an alternation between copulation and contact guarding. The duration of the final guarding in each pair during repeated mating was not included in (B).

On average, males copulated 10.08 times (SE = 3.77, min = 4, max = 17, *N* = 13). Separation was always voluntary by the male, with no observed female resistance. The average copulation duration was 6.74 min (SE = 4.04, min = 0.35 min, max = 14.04 min, *N* = 13) and copulation duration varied significantly among pairs (*χ* ^2^ = 13.18, *df* = 1, *p* < 0.001). The duration of copulation decreased significantly over the course of repeated copulations (β ± SE = −0.12 ± 0.02, *p* < 0.001; Figure 3A).

The average guarding duration was 13.61 min (SE = 35.87, min = 0.55 min, max = 277.02 min, *N* = 13). The duration of the final guarding period, defined as the time between the end of the final copulation and the pair's separation, averaged 67.43 min (SE = 82.67, min = 1.53 min, max = 277.02 min, *N* = 13) and was significantly longer than that of the other guarding periods (β ± SE = −1.65 ± 0.21, *p* < 0.001). Excluding the final guarding period, the average guarding duration was 7.43 min (SE = 17.02, min = 0.55 min, max = 141.08 min, *N* = 13) and the duration varied significantly among pairs (*χ* ^2^ = 29.56, *df* = 1, *p* < 0.001). Guarding duration, excluding the final guarding duration, increased significantly over repeated copulations and guarding periods (β ± SE = 0.09 ± 0.02, *p* < 0.001, Figure 3B).

Excluding the final guarding period, there was no significant correlation between copulation duration and guarding duration among pairs (among‐pair correlation (SE) = −0.27 (0.37)). However, within pairs, there was a significantly negative correlation between copulation duration and guarding duration (within‐pair correlation (SE) = −0.35 (0.09)). This indicates that during repeated copulation and guarding events, if one copulation event was longer, the subsequent guarding duration was shorter compared to other guarding periods.

### 3.4 Correlations Between Copulation Duration and Guarding Duration, and Morphological Traits

Males exhibiting longer average guarding durations had longer total foreleg lengths (Table 3, *p* = 0.039) and longer total hindleg lengths (Table 3, *p* = 0.025) compared with pronotum width. This is because the average guarding duration was positively correlated with foreleg femur length (Table 3, *p* = 0.031), foreleg tibia length (Table 3, *p* = 0.022), and hindleg femur length (Table 3, *p* = 0.022) relative to pronotum width. There was no correlation between the average guarding duration and the pronotum width, or the lengths of the siphon, forewing, or hindwing relative to the pronotum width.

**TABLE 3:** Correlations between morphological traits and mating behaviours of 14 males and 10 females.

	Morphological traits	Average single copulation duration	Average single guarding duration	Total copulation duration	Total guarding duration	Repeated copulation frequency
Male	Pronotum width	−0.30	0.36	−0.42	0.27	0.01
	Foreleg femur length	0.31	**0.60** *	−0.16	0.24	−0.43
	Foreleg tibia length	0.29	**0.63** *	0.12	0.42	−0.17
	Foreleg tarsus length	−0.04	0.36	−0.34	0.23	−0.20
	Foreleg total length^a^	0.29	**0.58** *	−0.16	0.22	−0.38
	Hindleg femur length	0.20	**0.66** *	−0.15	0.35	−0.31
	Hindleg tibia length	−0.14	0.53	−0.14	0.48	0.04
	Hindleg tarsus length	0.50	0.19	−0.03	−0.20	−0.40
	Hindleg total length^a^	0.24	**0.62** *	−0.11	0.30	−0.27
	Siphon length	0.47	0.33	0.08	0.19	−0.46
	Forewing length	−0.09	−0.15	−0.15	−0.06	−0.04
	Hindwing length	−0.27	−0.20	−0.29	−0.18	0.15
Female	Pronotum width	−0.50	−0.13	−0.03	0.22	0.28
	Foreleg femur length	0.10	−0.50	0.22	−0.45	0.10
	Foreleg tibia length	0.08	**−0.65** *	0.48	−0.58	0.34
	Foreleg tarsus length	0.49	−0.07	0.07	−0.15	−0.33
	Foreleg total length^a^	0.25	−0.47	0.26	−0.49	0.04
	Hindleg femur length	−0.22	**−0.64** *	0.17	−0.47	0.26
	Hindleg tibia length	−0.09	−0.31	0.26	−0.15	0.21
	Hindleg tarsus length	−0.2	−0.52	0.45	−0.10	0.52
	Hindleg total length^a^	−0.09	−0.58	0.39	−0.35	0.33
	Siphon length	0.2	−0.39	0.02	−0.37	−0.17
	Forewing length	−0.04	−0.27	0.45	−0.03	0.30
	Hindwing length	−0.39	−0.28	0.27	0.16	0.39

*Note:* All body component length data are relative to each individual's pronotum width. All morphological and behavioural traits were log‐transformed before analysis. Correlations (Spearman's rank correlation coefficients) that significantly differ from 0 are in bold.

^a^ Total leg length = the sum of the femur, tibia and tarsus lengths.

* *p* < 0.05.

Females who were guarded by males for longer average durations had shorter foreleg tibia lengths (Table 3, *p* = 0.043) and shorter hindleg femur lengths (Table 3, *p* = 0.047) relative to pronotum width. There was no correlation between average guarding duration and other leg appendage lengths, pronotum width, or lengths of the siphon, forewing, or hindwing relative to pronotum width (Table 3).

In both males and females, average copulation duration, total copulation duration (sum of copulation durations over repeated copulations), total guarding duration (sum of guarding durations over repeated copulations), and copulation frequency did not correlate with any morphological traits (Table 3).

**FIGURE 4:**
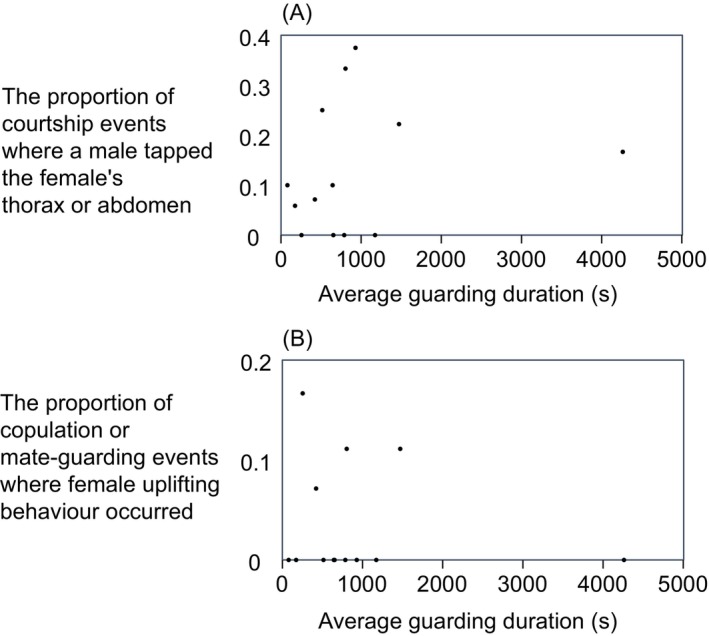
The relationship (A) between average guarding duration and male tapping behaviour or (B) between average guarding duration and female uplifting behaviour.

Furthermore, when we analysed the associations between the average guarding duration and other mating behaviours, no significant among‐pair correlations were found between the average guarding duration and the proportion of pre‐copulatory events with male tapping across repeated copulation (*R* = 0.29, *N* = 13, *t* = 1.01, *p* = 0.34, Figure 4A). Similarly, there were no significant correlations between the average guarding duration and the proportion of mate‐guarding events with female uplifting behaviour across repeated copulation (*R* = −0.01, *N* = 13, *t* = −0.03, *p* = 0.97, Figure 4B).

## 4 Discussion

We provide the first detailed description of the mating behaviour and repeated copulation and guarding patterns of *N. hoffmanni* . When *N. hoffmanni* male grasps a female and attempts to copulate, he typically engages in approximately 10 cycles of copulation and post‐copulation contact mate‐guarding until separation. Over these repeated copulations, the copulation duration decreased while the guarding duration increased. Additionally, the guarding duration correlated with both male and female morphological traits. Since a male grasps the female with his forelegs during mating, longer forelegs likely enhance the male's ability to hold the female effectively, resulting in prolonged average guarding durations. Our study thus highlights that a correlation between morphological traits and mating behaviour is strongly expected in insects engaging in prolonged contact mate‐guarding.

### 4.1 Repeated Copulation and Mate‐Guarding in *Nepa hoffmanni*

The decreased copulation duration of *N. hoffmanni* over repeated copulations might be due to the reduced amount of sperm in successive copulations. Males that engage in multiple copulations at short intervals are predicted to produce fewer sperm as copulation proceeds. For example, in the beetle *Tribolium castaneum* , the greatest amount of sperm is transferred during the first copulation, with the amount decreasing in successive copulations with females (Qazi, Herbeck, and Lewis 1996). Similarly, in the bedbug *Cimex lectularius* , the sperm vesicle volume decreases with successive copulations (Reinhardt, Naylor, and Siva‐Jothy 2011). Therefore, we suggest that the copulation duration in *N. hoffmanni* decreases due to the reduced amount of ejaculate in successive copulations with short intervals (guarding).

Additionally, the copulation duration was longest during the first copulation, which might be a strategy for maximising sperm transfer at the earliest stage of mating and enhancing fertilisation success under conditions of sperm competition. Given that a positive relationship has been found between copulation duration and the amount of sperm transferred in many insect species (e.g., Edvardsson and Canal 2006; Engqvist and Sauer 2003; Martin and Hosken 2002; Siva‐Jothy and Stutt 2003; reviewed in Simmons 2002), a longer copulation duration is the most effective way to ensure paternity. In *N. hoffmanni* , although females rarely resist physically during mating, they can control copulation by not opening their genital operculum. This means that females control access to copulation, so males need to transfer as much sperm as possible when given the opportunity. In addition, since *N. hoffmanni* females appear capable of expelling the male's sperm from their reproductive tract after copulation (H. Hyun, personal observation, Video S3), the best strategy for males to win sperm competition is to transfer a greater quantity of sperm over repeated copulations (e.g., Cordero, Santolamazza‐Carbone, and Utzeri 1995). Therefore, maximising the amount of sperm transferred during the first copulation can be the best strategy for males to increase their reproductive success. Moreover, if a female previously mated, transferring more sperm during the first copulation can help a male increase his fertilisation success against rival males. In the field cricket *Gryllus bimaculatus*, if a female retains the spermatophore of the first male for longer, the fertilisation success of the second male decreases (Simmons 1987). However, as the attachment duration of the spermatophore from the second male increases, the fertilisation success of the second male also improves (Simmons 1987). Therefore, the prolonged duration of the first copulation may have evolved as a reproductive strategy for *N. hoffmanni* males to increase their fertilisation success under sperm competition.

In contrast to the changes in copulation duration over repeated copulations, the duration of guarding increased. We hypothesise that *N. hoffmanni* might increase future reproductive investment and avoid excessive investment in current reproduction as copulation is repeated with the same female. If *N. hoffmanni* males invest a large amount of sperm early in mating, they can avoid transferring excess sperm to a female they have already copulated with several times, reserving sperm for potential future mates. This may cause the duration of copulation to decrease but the duration of guarding to increase with repeated copulations. Therefore, when *N. hoffmanni* males have already transferred sufficient sperm to females through repeated copulations, their reproductive efforts shift more toward post‐copulation mate‐guarding than toward prolonged copulation with the same female. This strategy enhances fertilisation success with the current female while preparing for future opportunities to mate and transfer sperm to another female.

In addition to the adaptive perspective on the increasing guarding duration and decreasing copulation duration with repeated copulation, such changes might simply be because males are unable to forage during mate‐guarding. This nutritional constraint may slow sperm replenishment, resulting in increased guarding duration (due to inefficient sperm replenishment) and decreased copulation duration (due to a smaller amount of sperm) with repeated copulations. In the beetle *Tribolium castaneum* , males who were starved before mating transferred fewer sperm and copulated for shorter durations than males fed before mating (Fedina and Lewis 2006). Similarly, in the wild tephritid fruit fly *Anastrepha fraterculus* , males fed a high‐protein diet stored more sperm and copulated longer (Abraham et al. 2011). In the ladybird *Adalia bipunctata* , males provided with insufficient food before mating copulated for shorter durations and transferred smaller ejaculates, but increased sperm density per ejaculate compared to that of well‐fed males (Perry and Rowe 2010).

These scenarios focus on the male perspective. From the female perspective, increased reluctance to mate over repeated copulations with the same male may explain the increasing guarding duration. According to our observations, the failure of male genitalia intromission in *N. hoffmanni* was mostly due to females refusing to open their genital operculum. In mating systems involving male–male scramble competition, it is common for females to resist males attempting to mate physically by kicking, pushing, shaking, or somersaulting (Arnqvist 1992; Blanckenhorn et al. 2000; Lauer 1996; Savalli and Fox 1999; Tsukamoto, Kuki, and Tojo 1994). In addition to direct resistance, females can also resist mating attempts by refusing to open their genitalia (e.g., Han and Jablonski 2009). *Nepa hoffmanni* females also exhibit such ‘indirect’ resistance toward males attempting to mate (H. Hyun, personal observation). Thus, increased guarding duration might reflect *N. hoffmanni* females' increased reluctance to mate with males attempting to copulate repeatedly. However, in our study, *N. hoffmanni* females were most reluctant to mate during the first mating attempt made by males and did not immediately open their genital operculum. Females were more likely to accept repeated mating attempts from males (H. Hyun, personal observation). Therefore, our observations of *N. hoffmanni* mating behaviour do not support the hypothesis that increased guarding duration during repeated copulations is due to female reluctance.

However, caution is needed when interpreting our results because the duration of copulation and mate‐guarding may be influenced by factors such as age, mating experience, and social conditions. In our study, *N. hoffmanni* individuals were collected from the wild during their mating season and maintained under laboratory conditions for several weeks to standardise their mating experience before the experiment. However, their prior mating experiences in the wild and the timing of their winter diapause termination could still have affected their behaviour. Additionally, we recorded their mating behaviour without same‐sex competitors and under an even sex ratio (1:1), which may differ from behaviours observed in the presence of competitors or under biased sex ratios. Thus, future studies should account for individual age and social experiences to provide a more comprehensive understanding of the mating behaviours of *N. hoffmanni* .

### 4.2 Correlations Between Morphological Traits and Mate‐ Guarding Behaviour

The correlation between body component length and guarding duration is notable in animal species where males engage in contact guarding by grasping resistant females with their legs. In the toad *Bufo terrestris* , males guarding mates during the breeding season had longer forelimbs than did solitary males, independent of the body length (Lee 1986). In the Harlequin toad *Atelopus longissimus*, males guarding females had broader forelimbs and exerted stronger clasping forces than did solitary males (Rueda‐Solano et al. 2022). In the diving beetle *Acilius japonicus* , males with larger suction cups on their forelegs had a greater probability of successful mounting and copulation (Kiyokawa and Ikeda 2022). Additionally, in the water strider *G. gracilicornis* , males produce the strongest grasping force during the precopulation stage when their body length is 86% of the female's body length, resulting in size‐assortative mating (Han and Jablonski 2018; Han et al. 2010). Therefore, in species where males must tightly hold females during mating, the morphological characteristics of body components, such as length, play a crucial role in influencing mating and guarding duration, thereby determining reproductive success.

We found that the leg lengths of *N. hoffmanni* males were positively associated with the average guarding duration, suggesting that longer legs may enhance grasping efficiency and increase guarding duration. However, given the lack of correlation between leg length and total guarding duration over repeated copulations, it is unlikely that longer legs are associated with increased grasping efficiency. Additionally, since *N. hoffmanni* females do not exhibit vigorous resistance behaviours to dislodge mounting males (H. Hyun, personal observation), grasping efficiency is unlikely to be a significant factor in determining the duration of guarding by *N. hoffmanni* males. We also observed that males always dismounted voluntarily when the pair was separated (H. Hyun, personal observation). Therefore, the guarding duration of *N. hoffmanni* is unlikely to be related to how effectively and tightly males grasp females during the guarding period.

What, then, accounts for the positive association between male leg length and guarding duration in *N. hoffmanni* ? Considering that *N. hoffmanni* males frequently tap females using their legs during mate‐guarding, one possibility is that males with shorter legs might effectively stimulate females and induce them to open their genital operculum more rapidly. This could lead to shorter guarding durations and quicker initiation of the next copulation. Pre‐copulatory stimulation by males is known to reduce female mating resistance, and the effectiveness of such stimulation may depend on male morphological characteristics. For example, in the bushcricket *Metrioptera roeselii* , female resistance was reduced when males stimulated females symmetrically using a pair of titillators during copulation, in contrast to when males produced asymmetrical stimulation with an experimentally manipulated single titillator (Wulff and Lehmann 2016). Similarly, if tapping by males with shorter forelegs effectively relaxes females and stimulates them to open their genital operculum, males with shorter legs could shorten the guarding duration and achieve new copulations more quickly.

In addition, in species where males grasp females during guarding, female leg morphology may also correlate with guarding duration if females resist mating with their legs. We found that female *N. hoffmanni* with longer foreleg tibias and longer hindleg femurs experienced shorter guarding durations. Although *N. hoffmanni* females did not exhibit vigorous resistance (H. Hyun, personal observation), they sometimes shook, lifted their bodies, and moved around while carrying males during the guarding period. Such behaviours might make it difficult for males to maintain a stable grasp or expose them above the water surface. In this situation, mounting males may try to copulate again quickly before dismounting. Thus, females with longer legs may move more frequently during the guarding period, prompting mounting males to stop guarding and attempt another copulation sooner.

We suggested that the association between leg length and efficiency of behaviour during guarding periods (e.g., male tapping behaviour or female resistance) might explain the relationship between leg length and guarding duration in both sexes. However, our results showed no relationship between guarding duration and male tapping behaviour or between guarding duration and female uplifting behaviour (Figure 4). Alternatively, mate choice might explain the relationship between leg length and guarding duration. Females may prefer males with shorter legs, and males may prefer females with longer legs, leading to shorter guarding durations. However, it is difficult to explain why leg length is a target of mate choice. Due to the small sample size, we cannot draw strong conclusions about the relationship between leg length and guarding duration. Therefore, in future studies, more samples should be collected to test the relationship between leg length, tapping behaviour, and guarding duration.

### 4.3 Conclusion

Our study is the first to report on the detailed mating behaviour of *N. hoffmanni* . We observed the entire mating process, from initial physical contact to pair separation, detailing their behaviour. We found that *N. hoffmanni* males engage in repeated copulation and mate‐guarding while holding females. We suggest that sperm competition may cause a decrease in copulation duration and an increase in guarding duration over repeated copulations. Additionally, we found that males with longer legs and females with shorter legs were associated with longer mate‐guarding durations. However, our study was limited in its ability to control for the age and mating experience of *N. hoffmanni* . Future studies should address these limitations by using offspring of wild‐collected individuals to control for age and social experience. In addition, further studies should investigate mating behaviours under different social conditions, such as varying sex ratios and population densities, to provide a more comprehensive understanding of the factors affecting mating dynamics in this species.

## Author Contributions

**Hyoseul Hyun:** conceptualization (equal), data curation (equal), formal analysis (equal), investigation (equal), writing – original draft (equal), writing – review and editing (equal). **Byeongho Lee:** formal analysis (equal), investigation (equal). **Chang S. Han:** conceptualization (equal), data curation (equal), formal analysis (equal), funding acquisition (equal), investigation (equal), supervision (equal), writing – original draft (equal), writing – review and editing (equal).

## Conflicts of Interest

The authors declare no conflicts of interest.

## Supporting information


Appendix S1

Video S1

Video S2

Video S3

## Data Availability

All data is included as a Supporting Information.
